# Supplementary material for: The breast pre-cancer atlas illustrates the molecular and micro-environmental diversity of ductal carcinoma in situ
Source: NPJ Breast Cancer. 2022 Jan 13;8:6. doi: 10.1038/s41523-021-00365-y (PMC8758681; doi:10.1038/s41523-021-00365-y)
Supplement: Supplementary file 1 — Supplementary Data 2 [file 41523_2021_365_MOESM1_ESM.pdf]

Supplementary Data 2

This supplementary dataset includes all supplementary figures cited in the article.

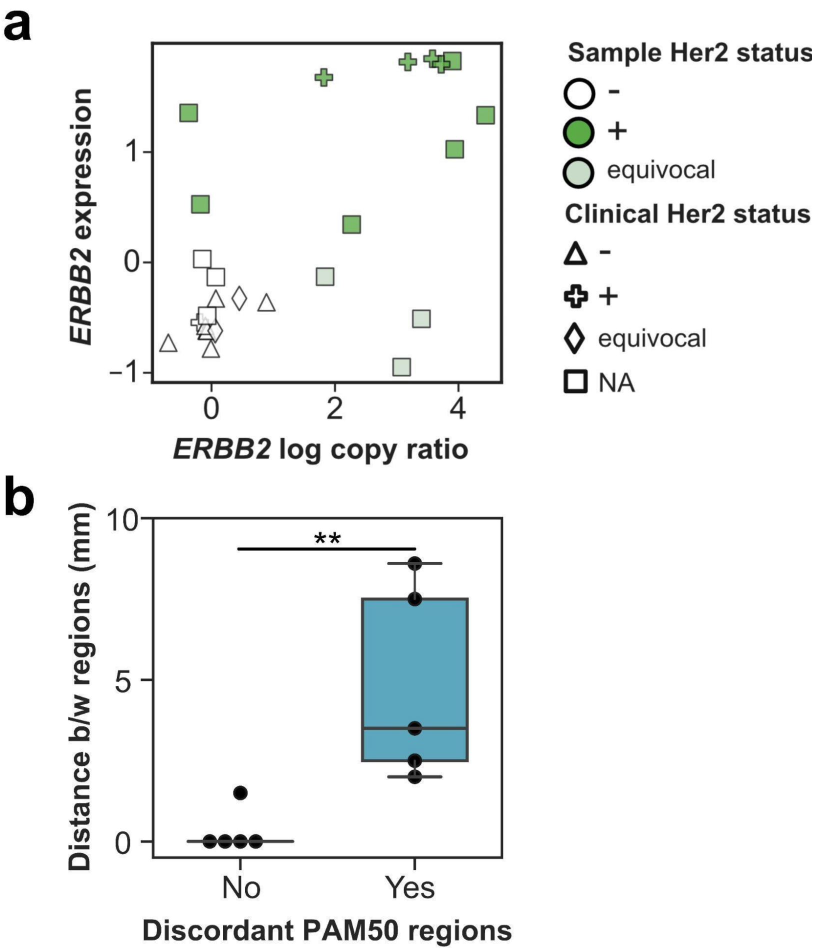

Supplementary Figure 1. Pure DCIS characterization. (a) Estimation of Her2 status. The DNA-based log_2 copy number ratio (x-axis) and RNA-based expression level (y-axis) of *ERBB2* gene are displayed for 26 samples with both data available. **(b)** PAM50 discordance between regions in relationship with spatial distance between regions, **p<0.01, Mann-Whitney U test.

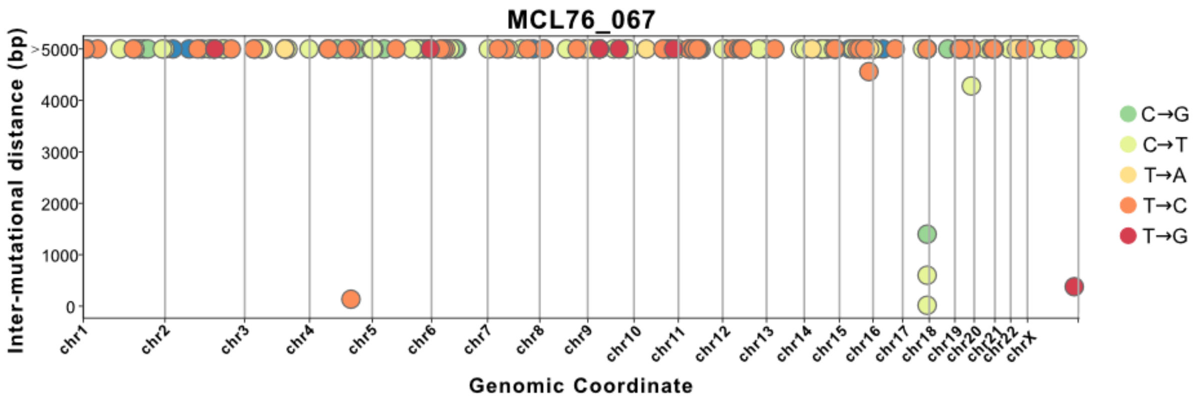

Supplementary Figure 2. Likely kataegis event in MCL76_067_16600 in chromosome 17. Along the genome coordinate (x-axis), the relative distance between proximal mutations (y-axis) as well as their substitution type (colors) are indicated.

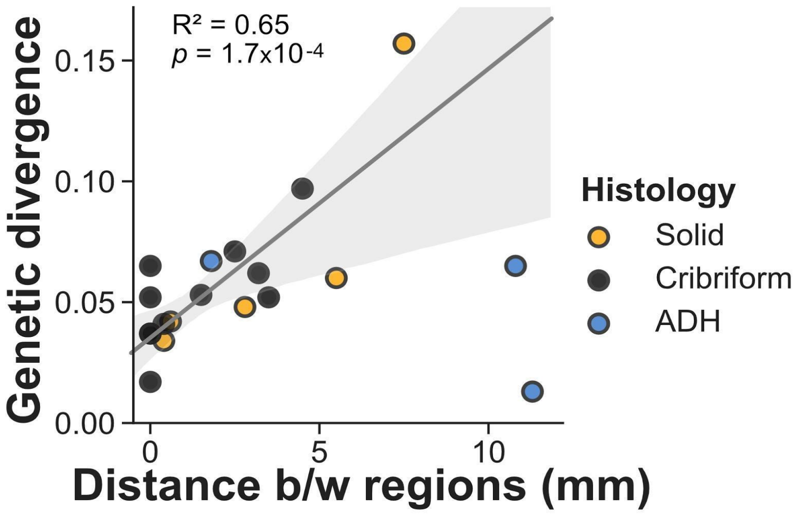

Supplementary Figure 3. Genetic divergence in multi-region DCIS. CNA-based genetic divergence (y-axis) of each pair of histologically concordant regions (dot) as a function of the minimum physical distance between them (x-axis), colored by their histology. Linear regression line fit of DCIS samples shown in solid dark gray line, with 95% confidence interval estimate based on bootstrapping in light gray.

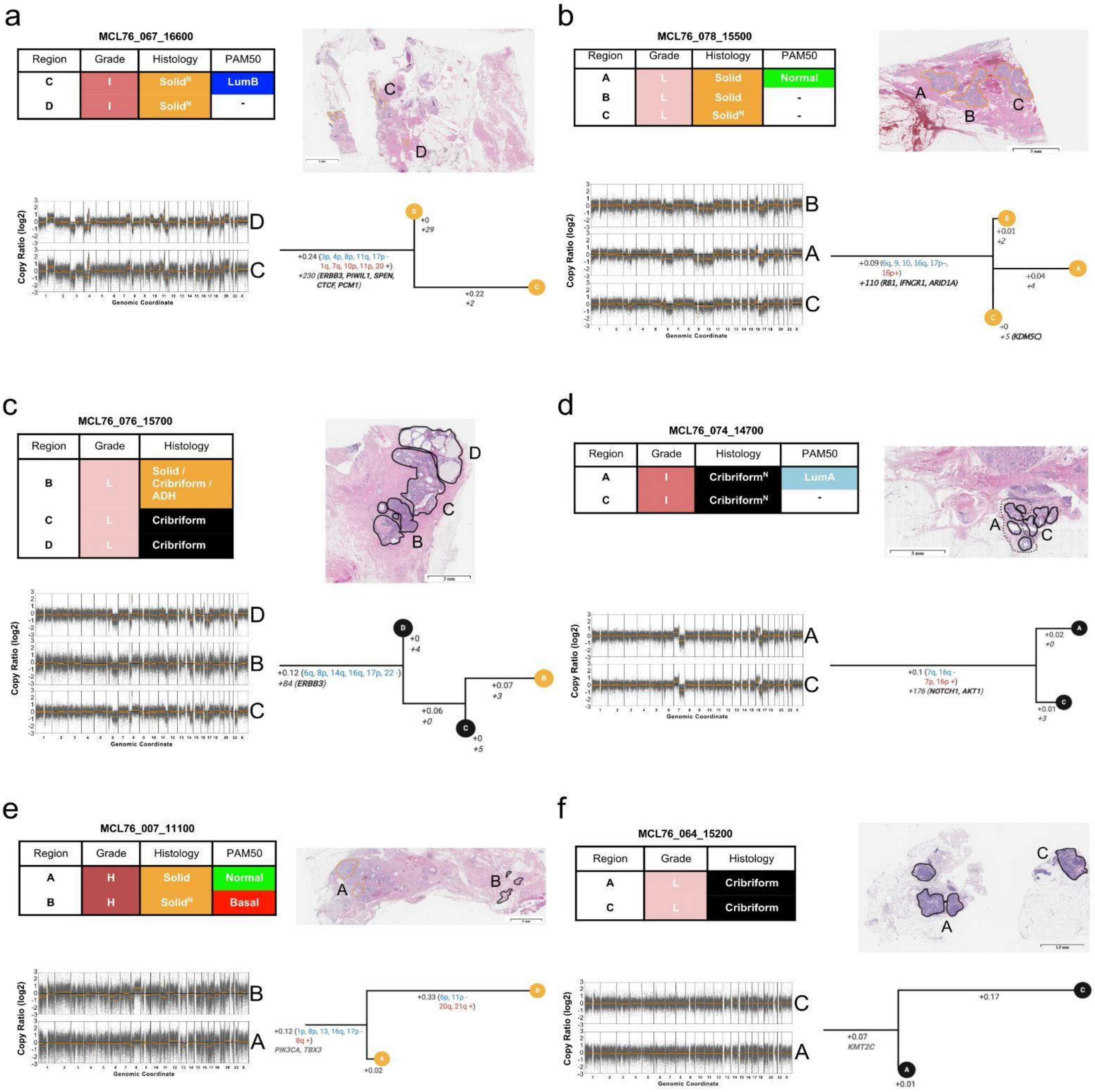
a
MCL76_067_16600
Region | Grade | Histology | PAM50
C | I | SolidN | LumB
D | I | SolidN | -
C
D
Copy Ratio (log2)
Genomic Coordinate
+0.24 (3p, 4p, 8p, 11q, 17p - 1q, 7q, 10p, 11p, 20 +)
+230 (ERBB3, PIWIL1, SPEN, CTCF, PCM1)
+0
+29
+0.22
+2
b
MCL76_078_15500
Region | Grade | Histology | PAM50
A | L | Solid | Normal
B | L | Solid | -
C | L | SolidN | -
A
B
C
+0.09 (6q, 9, 10, 16q, 17p-, 16p+)
+110 (RB1, IFNGR1, ARID1A)
+0.01
+2
+0.04
+4
+0
+5 (KDM5C)
c
MCL76_076_15700
Region | Grade | Histology
B | L | Solid / Cribriform / ADH
C | L | Cribriform
D | L | Cribriform
D
B
C
+0.12 (6q, 8p, 14q, 16q, 17p, 22 -)
+84 (ERBB3)
+0
+4
+0.07
+3
+0.06
+0
+0
+5
d
MCL76_074_14700
Region | Grade | Histology | PAM50
A | I | CribriformN | LumA
C | I | CribriformN | -
A
C
+0.1 (7q, 16q - 7p, 16p +)
+176 (NOTCH1, AKT1)
+0.02
+0
+0.01
+3
e
MCL76_007_11100
Region | Grade | Histology | PAM50
A | H | Solid | Normal
B | H | SolidN | Basal
A
B
+0.12 (1p, 8p, 13, 16q, 17p - 8q +)
PIK3CA, TBX3
+0.33 (6p, 11p - 20q, 21q +)
+0.02
f
MCL76_064_15200
Region | Grade | Histology
A | L | Cribriform
C | L | Cribriform
C
A
+0.07
KMT2C
+0.17
+0.01

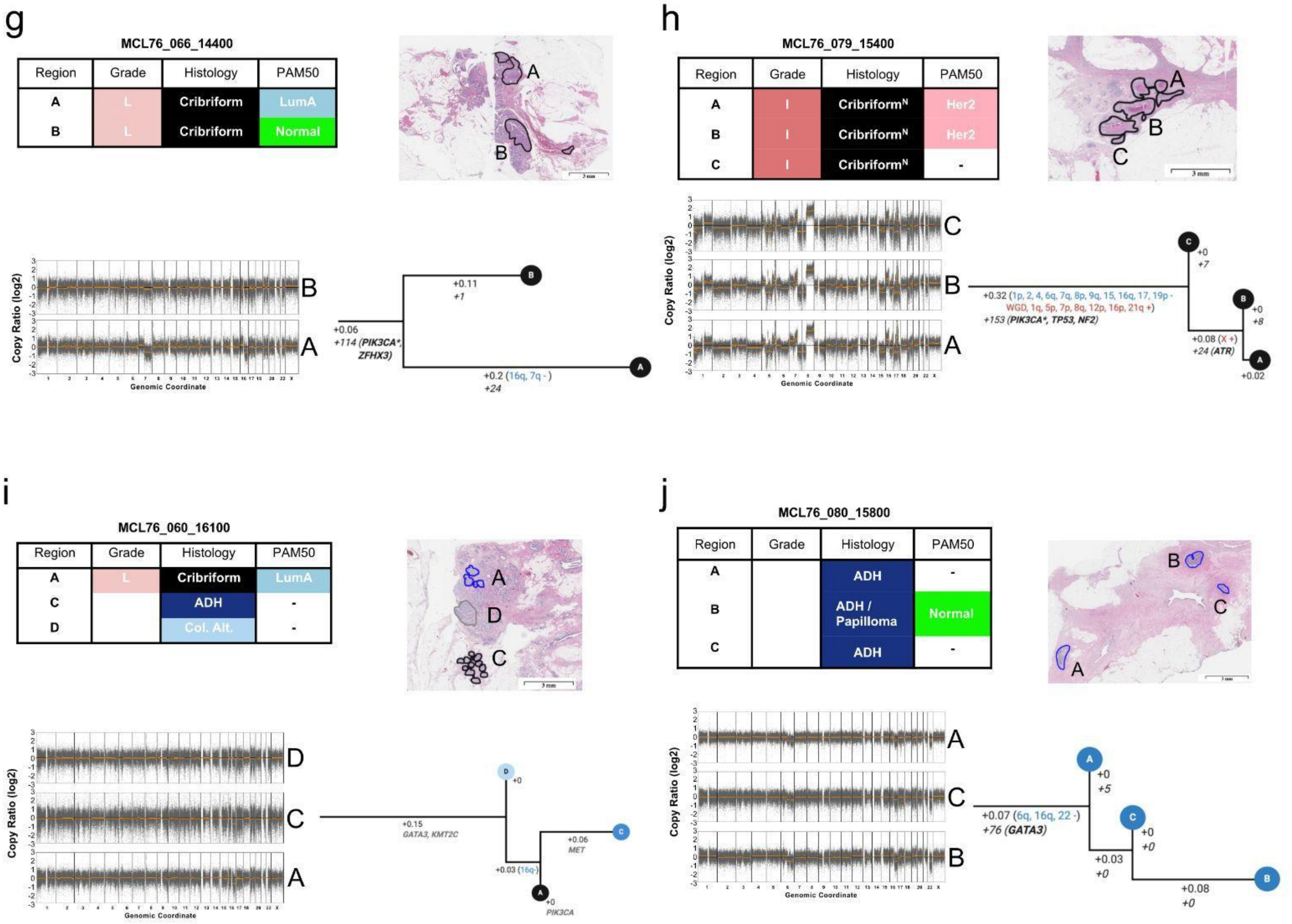

Supplementary Figure 4. Phylogenetic trees for multi-region DCIS samples. (a-j) Clonal reconstruction using CNA and somatic mutations in 25 related regions across 10 samples. In each panel, _top left:_ a table describing the name, nuclear grade and histological architecture of each region in a sample is shown, (necrosis is indicated with N); _top right:_ shows an H&E image of the sample with dissected regions drawn on the image; _bottom left_: Copy number profiles for each region in the sample, genomic coordinates are indicated on the X-axis and the $\log_2$ copy ratio on the Y-axis. Bins are indicated in dark-grey and segments in orange; _bottom right_: Phylogenetic tree for the sample with leaf nodes indicating a single dissected region colored by histology. The tree is rooted to a normal diploid ancestor. Branch lengths are hamming distances based on CNA segments. Branches are labeled with 1) CNA-based branch length, and, when available, 2) arm-level CNA losses (blue) and gains (red) and 3) somatic mutation number (italic) with mutations in breast cancer driver genes indicated (black: high coverage, grey: low coverage). CNA smaller than arms, or on driver genes are not displayed.

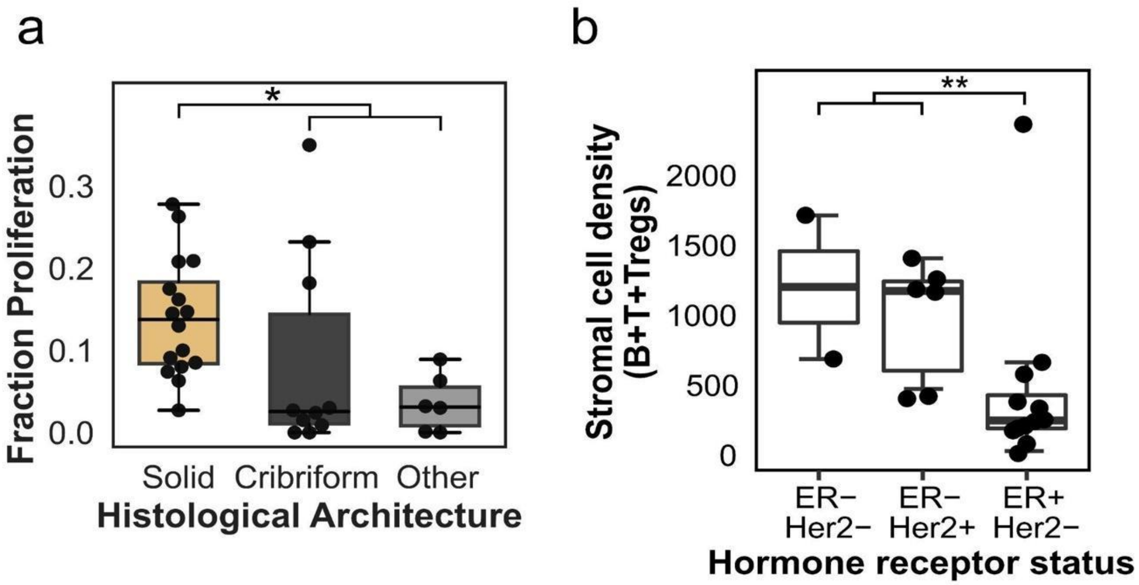

Supplementary Figure 5. (a) Estimates of the fraction of Ki67+ cytokeratin positive cells in epithelium of the mIHC images according to each histological architecture. *p<0.05, Mann-Whitney U test. **(b)** Stromal immune cell density differences between DCIS subtypes. B=B-cells, T=T-cells and Tregs=Regulatory T-cells. **p<0.01, Mann-Whitney U test.

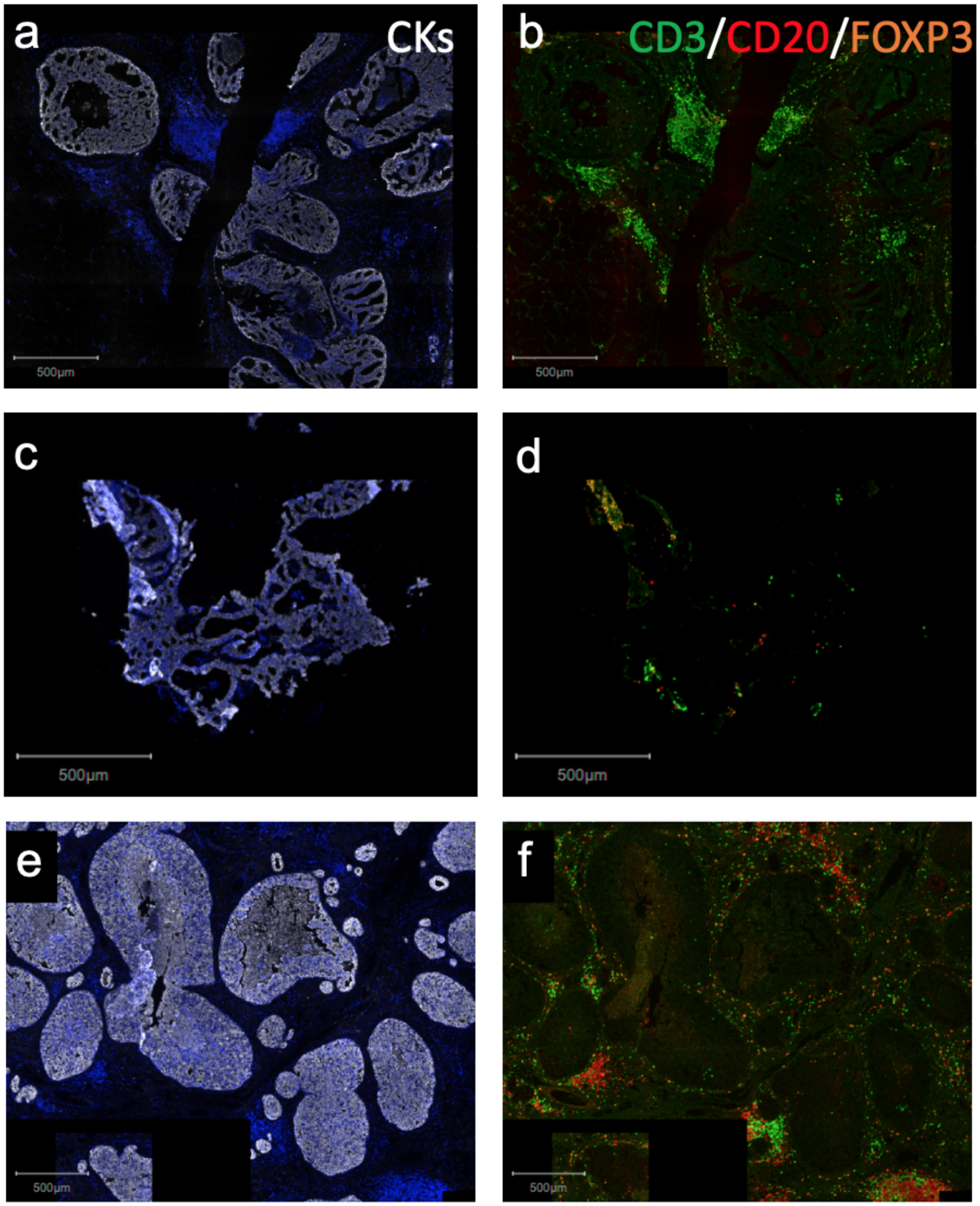

Supplementary Figure 6. Multiplex immuno-fluorescent images representative of the three immune-states. The pan-cytokeratin (white) and nuclear (DAPI - blue) staining (a,c,e) and the matching CD3 (T-cells, green), CD20 (B-cells, red) and FOXP3 (T-reg, orange) stainings (b,d,f) are shown for specimen representative of the Active (a,b, MCL78_013_10001), Suppressed (c,d, MCL76_049_18100) and Excluded (e,f,MCL76_074_14700) states.

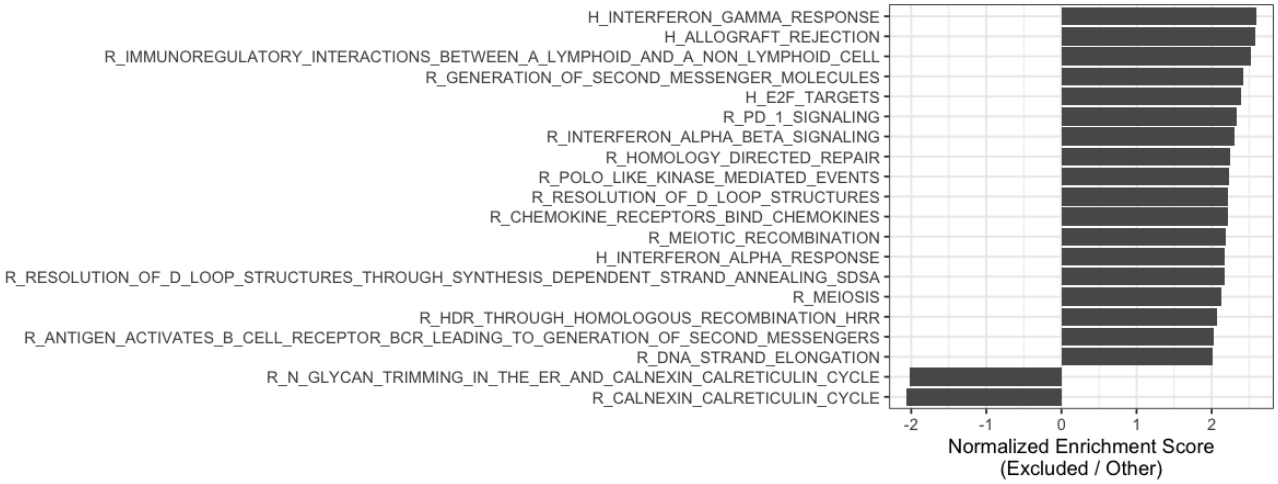

Supplementary Figure 7. Gene sets significantly deregulated in epithelium of regions in the Excluded immune state. All Hallmark (H) and Reactome (R) genesets were tested. Genesets with an absolute normalized enrichment score greater than 2 and with FDR less than 0.05 are represented.

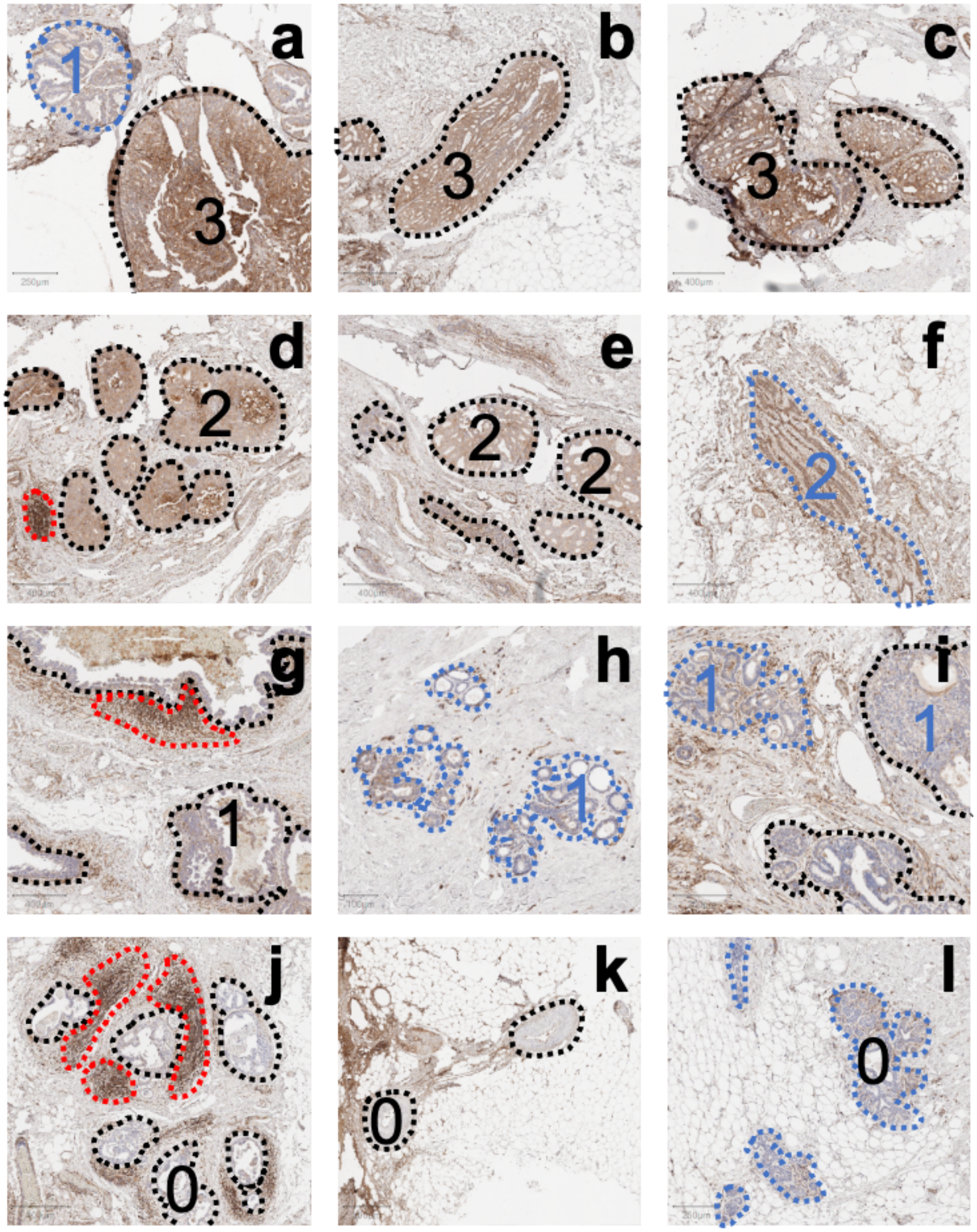

Supplementary Figure 8. MHC1 immunostaining scoring. Selected areas of DCIS (black) or normal ducts (blue) are indicated together with their associated expression score from 3 (panel a-c), 2 (d-f), 1 (g-i), 0 (j-l). Additional MHC1 high lymphocytes areas are indicated in red. Scale bar indicated in bottom left.
