# Supplementary material for: The breast pre-cancer atlas illustrates the molecular and micro-environmental diversity of ductal carcinoma in situ
Source: NPJ Breast Cancer. 2022 Jan 13;8:6. doi: 10.1038/s41523-021-00365-y (PMC8758681; doi:10.1038/s41523-021-00365-y)
Supplement: Supplementary file 3 — Reporting Summary [file 41523_2021_365_MOESM3_ESM.pdf]

nature portfolio

Corresponding author(s): Olivier Harismendy

Last updated by author(s): Nov 17, 2021

Reporting Summary

Nature Portfolio wishes to improve the reproducibility of the work that we publish. This form provides structure for consistency and transparency in reporting. For further information on Nature Portfolio policies, see our Editorial Policies and the Editorial Policy Checklist.

Statistics

For all statistical analyses, confirm that the following items are present in the figure legend, table legend, main text, or Methods section.

n/a	Confirmed	
☐	☒	The exact sample size (n) for each experimental group/condition, given as a discrete number and unit of measurement
☐	☒	A statement on whether measurements were taken from distinct samples or whether the same sample was measured repeatedly
☐	☒	The statistical test(s) used AND whether they are one- or two-sided<br>*Only common tests should be described solely by name; describe more complex techniques in the Methods section.*
☐	☒	A description of all covariates tested
☐	☒	A description of any assumptions or corrections, such as tests of normality and adjustment for multiple comparisons
☐	☒	A full description of the statistical parameters including central tendency (e.g. means) or other basic estimates (e.g. regression coefficient) AND variation (e.g. standard deviation) or associated estimates of uncertainty (e.g. confidence intervals)
☐	☒	For null hypothesis testing, the test statistic (e.g. F, t, r) with confidence intervals, effect sizes, degrees of freedom and P value noted<br>*Give P values as exact values whenever suitable.*
☒	☐	For Bayesian analysis, information on the choice of priors and Markov chain Monte Carlo settings
☒	☐	For hierarchical and complex designs, identification of the appropriate level for tests and full reporting of outcomes
☒	☐	Estimates of effect sizes (e.g. Cohen's d, Pearson's r), indicating how they were calculated

Our web collection on statistics for biologists contains articles on many of the points above.

Software and code

Policy information about availability of computer code

Data collection	Scanned multispectral images were unmixed on inForm software (ver.2.4.0, Akoya Bioscience). Alternatively, QuPath software 2.3.1 was also used to perform similar imaging analysis on unmixed images converted to multi-layered TIFF format by inForm software
Data analysis	Sequencing data was analyzed using bcbio-nextgen (v1.1.6) as a workflow manager [20]. Adapter sequences were trimmed using Atropos (v1.1.22), the trimmed reads were subsequently aligned with bwa-mem (v0.7.17) to reference genome hg19, then PCR duplicates were removed using biobambam2 (v2.0.87) 66–68. Additional BAM file manipulation and collection of QC metrics was performed with picard (v2.20.4) and samtools (v1.9). Single nucleotide variants (SNVs) and short insertions and deletions (indels) were called with VarDictJava (v1.6.0), and Mutect2 (v2.2). Multi-region segmentation was performed with the R CopyNumber (v1.26.0) package. Treeomics (v1.7.10) was run with the default parameters except for e=0.02.

For manuscripts utilizing custom algorithms or software that are central to the research but not yet described in published literature, software must be made available to editors and reviewers. We strongly encourage code deposition in a community repository (e.g. GitHub). See the Nature Portfolio guidelines for submitting code & software for further information.

Data

Policy information about availability of data

All manuscripts must include a data availability statement. This statement should provide the following information, where applicable:

- Accession codes, unique identifiers, or web links for publicly available datasets
- A description of any restrictions on data availability
- For clinical datasets or third party data, please ensure that the statement adheres to our policy

The raw RNA and DNA sequencing data has been deposited in dbGAP phs002225. High resolution whole slide images of the H&E stains and corresponding

March 2021

annotations can be viewed on the JPL LabCAS portal (digital object identifiers included in the Supplementary Table 1). Images corresponding to the stitched field of views of the region of interest in the multiplex immunohistochemistry are made available as multilayered tiff files on the JPL LabCAS portal https://doi.org/10.48577/rrry-pj94 (UVM) and https://doi.org/10.48577/3gns-rn74 (UCSD).

Field-specific reporting

Please select the one below that is the best fit for your research. If you are not sure, read the appropriate sections before making your selection.

☒ Life sciences ☐ Behavioural & social sciences ☐ Ecological, evolutionary & environmental sciences

For a reference copy of the document with all sections, see nature.com/documents/nr-reporting-summary-flat.pdf

Life sciences study design

All studies must disclose on these points even when the disclosure is negative.

Sample size	no sample size calculation was perfromed. The study is observational by design and 40 specimen capture sufficient variability in many histological and molecular features as determined from previous studies.
Data exclusions	Data exclusion was limited to lack of data. Lack of data can be due to sample, primary or secondary failing quality controls
Replication	The data is derived from patients specimen, which are by definition unique. In a few instances, matching regions were micro-dissected in sequential specimen section and analyzed jointly, before merging the results.
Randomization	not applicable
Blinding	not applicable

Reporting for specific materials, systems and methods

We require information from authors about some types of materials, experimental systems and methods used in many studies. Here, indicate whether each material, system or method listed is relevant to your study. If you are not sure if a list item applies to your research, read the appropriate section before selecting a response.

Materials & experimental systems

n/a	Involved in the study	
☐	☒	Antibodies
☒	☐	Eukaryotic cell lines
☒	☐	Palaeontology and archaeology
☒	☐	Animals and other organisms
☐	☒	Human research participants
☒	☐	Clinical data
☒	☐	Dual use research of concern

Methods

n/a	Involved in the study	
☒	☐	ChIP-seq
☒	☐	Flow cytometry
☒	☐	MRI-based neuroimaging

Antibodies

Antibodies used	Antibodies used in mIHC are anti-CD3 (clone 2GV6, Ventana), anti-CD20 (clone L26, Ventana), anti-Ki67 (clone 30-9, Ventana), anti-FOXP3 (clone SP97, Spring), anti-pan cytokeratin (CK; clone AE1/AE3, DAKO), anti-CD117 (clone c-kit, DAKO)
Validation	from DAKO: "The FLEX RTU Solution offers dedicated series of high-quality, pre-diluted, ready-to-use (RTU) primary antibodies that are accompanied by appropriate validated protocols to support your lab in providing reliable and reproducible diagnostic results.<br><br>The robust IHC tests are calibrated and validated for reliable diagnostic use, ensuring that the antigen is correctly demonstrated at both high and low expression levels in tissue to support your lab in reducing the risk of false negative and false positive results.<br><br>The GA-Series FLEX RTU primary antibodies listed in this section are packaged in Dako Omnis vials for use on Dako Omnis instruments and can only be used with the EnVision FLEX system for Dako Omnis."

Human research participants

Policy information about studies involving human research participants

Population characteristics	Eligibility criteria were: 1) adult female, 2) pure DCIS diagnosis (without evidence of invasive disease), 3) with available pathology blocks.

Recruitment	the study was retrospective by desiggn
Ethics oversight	University of Vermont Institutional Review Board. University of California San Diego Institutional Review Board. Bot IRB waived the requirement for informed consent given the retrospective nature of the study.

Note that full information on the approval of the study protocol must also be provided in the manuscript.
